# Hyaluronic Acid-Based Drug Delivery Systems for Cancer Therapy

**DOI:** 10.3390/cells14020061

**Published:** 2025-01-07

**Authors:** Ekaterina Pashkina, Maria Bykova, Maria Berishvili, Yaroslav Lazarev, Vladimir Kozlov

**Affiliations:** 1 Research Institute of Fundamental and Clinical Immunology, 14, Yadrintsevskaya St., 630099 Novosibirsk, Russia; 2 Department of Clinical Immunology, Novosibirsk State Medical University, 52, Krasny Prospect, 630091 Novosibirsk, Russia; 3 Faculty of Natural Sciences, Novosibirsk State University, 2, Pirogova Street, 630090 Novosibirsk, Russia

**Keywords:** hyaluronic acid, extracellular matrix, drug delivery system, nanoparticle, cancer therapy, supramolecular system

## Abstract

In recent years, hyaluronic acid (HA) has attracted increasing attention as a promising biomaterial for the development of drug delivery systems. Due to its unique properties, such as high biocompatibility, low toxicity, and modifiability, HA is becoming a basis for the creation of targeted drug delivery systems, especially in the field of oncology. Receptors for HA overexpressed in subpopulations of cancer cells, and one of them, CD44, is recognized as a molecular marker for cancer stem cells. This review examines the role of HA and its receptors in health and tumors and analyzes existing HA-based delivery systems and their use in various types of cancer. The development of new HA-based drug delivery systems will bring new opportunities and challenges to anti-cancer therapy.

## 1. Introduction

One of the most pressing problems currently facing pharmacology is the development of new chemotherapeutic drugs. The proportion of malignant tumors sensitive to chemotherapy is quite small. Alternative treatments, such as surgery and radiation therapy, are not always effective, especially at the metastasis stage. Cytostatic drugs often have many side effects, including nephrotoxicity, polyneuropathy, myelosuppression, ototoxicity, allergic reactions, dyspepsia, and cardiovascular disorders. The development of drug delivery systems is aimed at improving the effectiveness of the drug without increasing side effects [1,2].

One of the modern and actively developing areas at the intersection of chemistry, pharmacology, and oncology is supramolecular chemotherapy, based on the possibility of forming complexes of the “host-guest” type [1,2,3,4]. Macrocyclic compounds such as cucurbiturils, cyclodextrins, calixarenes, etc., are ideal platforms for constructing chemotherapeutic platforms through supramolecular interactions, since they contain cavities of controlled size into which “guest” molecules can penetrate [5,6]. Supramolecular chemotherapy, which combines non-covalent interactions and traditional chemotherapy, has demonstrated a number of advantages, for example, the ability to increase the solubility and stability of drugs and reduce side effects, and it can also be used for targeted drug delivery. As is known, the essence of targeted delivery is that the drug itself, and more often the means of its delivery (vector, container) are modified by molecules that recognize structures on target cells. In the case of supramolecular delivery systems, hyaluronic acid can both serve as a ligand and act as a molecular framework [7,8]. Since hyaluronic acid is not a bioinert molecule, when creating drug delivery systems based on hyaluronic acid, it is necessary to take into account its role under normal conditions and in oncology.

## 2. Hyaluronic Acid

Hyaluronic acid (HA) is a natural polysaccharide that is widespread in the human body and is of great importance for its physiological processes [9]. HA is one of the key components of the extracellular matrix and is found in various tissues and structures, mainly in connective tissue, joints, epidermis, and the eyeball. Due to its high hydrophilicity, HA has unique properties, such as the ability to form viscous gel-like structures, retain moisture, provide lubrication of joints, improve elasticity and firmness of the skin and tissue hydration, and actively participate in the regeneration of damaged tissues [10,11,12].

**Figure 1:**
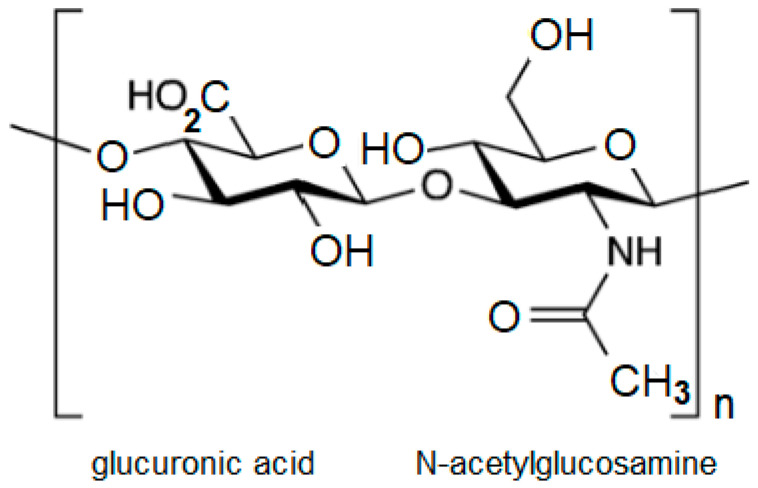
The structure of hyaluronic acid.

Hyaluronic acid is a glycosaminoglycan formed by repeating dimers of the disaccharide N-acetylglucosamine and glucuronic acid (Figure 1). The unique characteristic of this polysaccharide is its lack of association with protein structures and the absence of sulfated groups, which distinguishes it from other glycosaminoglycans.

Hyaluronic acid (HA) is an important component of the intercellular space of many tissues in the body. In addition to its reinforcing and buffering functions, HA is an important source of information about the environment for cells. The key factors in this information are the number of cellular contacts with HA via HA-binding receptors, as well as the length of the bound hyaluronic acid fragment [13,14].

The most important information from the environment for a cell is information about the integrity of the tissue of which it is a part. The integrity of the tissue, and, therefore, the components of the extracellular matrix (ECM), which include HA, can be disrupted during wound injury, inflammation, and tissue remodeling. During these effects, the number of cell contacts with the ECM decreases, and the ECM itself can be destroyed with the formation of oligomers [15,16,17]. Both of these factors inform the cell of the need for an adaptive response to the current conditions, which on a tissue scale is expressed in its remodeling. Namely, this manifests in the proliferation of cellular components, increased production of ECM, testing of tissue cells for “professional suitability” (suitability for proliferation), migration of specialized cells to control and restore tissue function, as well as renewal of vascularization of new tissue [13,18].

HA is a ligand for such receptors as CD44, RHAMM (receptor for hyaluronic acid-mediated motility), lymphatic vessel endothelial hyaluronan receptor 1 (LYVE1), hyaluronan receptor for endocytosis (HARE/stabilin-2), LAYN, and Toll-like receptors (TLRs) [19,20]. The interaction of HA with the CD44 receptor activates key intracellular signaling pathways, which increases the expression of several genes responsible for proliferation and cell survival. This interaction also induces the reorganization of the actin cytoskeleton, which leads to active cell migration.

Tumors, as a pathological type of tissue remodeling process, use the same logic and signaling pathways for tissue degeneration. The role of HA in oncology is based mainly on data from experiments on the CD44 receptor regulation system [13,21,22]. It is expressed in many cells in the body, and its regulation is often of decisive importance in in vivo experiments in animal models. Nevertheless, there are tumors that are CD44-dependent, CD44-weakly-dependent, or CD44-independent, such as Ewing’s sarcoma. Other HA-binding receptors may also come to the fore in the regulation of such tumors. Data on their isolated and synergistic effect with CD44 were obtained on cell cultures.

In the literature, it is common to divide HA chains into functional groups depending on the length of the polysaccharide: oligo-HA (<10 kDa), low molecular weight, 10–200 kDa (L-HA), medium molecular weight, 0.25–1 MDa (M-HA), high molecular weight, more than 1 MDa (H-HA), and HA with very high molecular weight (VH-HA, >6000 kDa) [18].

The interaction of receptors with HA depends on the molecular weight of the HA (Table 1). HA with a higher molecular weight has high avidity for CD44 and can lead to its clustering on the membrane surface, thereby modulating the cellular response. H-HA can form steric protection on the membrane surface, limiting access to cell death receptors, and protecting cells from apoptosis [14,18].

Low- and medium-molecular-weight HA stimulates the synthesis of heat shock proteins, demonstrating antiapoptotic, pro-angiogenic, and immunostimulating properties [13,14,18,21]. Experiments demonstrate that L-HA has a pro-inflammatory effect and can activate mouse alveolar macrophages or cause phenotypic maturation of human dendritic cells. It was found that L-HA increases the expression of cytokines such as MMP-12, plasminogen activator inhibitor-1, macrophage inflammatory protein (MIP)-1α, MIP-1β, monocyte chemoattractant-1, keratinocyte chemoattractant, interleukin (IL)-8, and IL-12 in macrophages. Stimulation of CD44 by L-HA leads to the activation of two tyrosine kinases. Activation of p185HER2 promotes cell growth, while c-Src kinase activity results in the phosphorylation of cytoskeletal proteins, thereby inducing cell migration.

Dong et al. [23] suggested that the pro-inflammatory properties of L-HA and oligo-HA were due to the contamination of reagents and glassware with endotoxins. For example, with pro-inflammatory agents such as LPS, its contamination at 5 pg/mL is sufficient to induce detectable levels of inflammatory cytokines by cells.

In another work, it was shown that oligo-HA reduces the expression of mRNA TNF-α, IL-6, IL-1β, and IFN-β and the production of TNF-α IFN-β proteins. Enzymatically hydrolyzed 2-mer HA inhibited LPS-induced lung inflammation in mice [24]. M-HA inhibits the synthesis of NO in LPS-activated macrophages and increases the expression of TGF-b1, IL-10, IL-11, and Arg1 in non-activated macrophages. But M-HA also enhanced the expression of mRNA Il-1b, and CCL2 in LPS-activated macrophages (relative to LPS-activated macrophages without HA) [25].

**Table 1:** Characteristics of HA with different molecular weights.

Weight in kDa	Dosage	Physiological Effects	Receptors	References
1.516 kDa	40 μg/mL	HA activates NF-kB via TLR2 and TLR4 and increases the expression of MMP-13, TNF, IL-1b, MMP-13, iNOS. HA oligosaccharides are unable to communicate with CD44.	TLR2; TLR4	[26]
0.758 kDa	10 μg/mL	HA is a potential antagonist of TLR4. HA reduces the expression of TNF, IL-6, IL-1β, IFN-β mRNAs and the production of TNF-α IFN-β proteins. Enzymatically hydrolyzed 2-mer HA inhibited LPS-induced lung inflammation in mice.	TLR4	[24]
<200 kDa	25–500 μg/mL	HA induces the production of β-defensin 2 (DEFB2) in keratinocytes, but not IL-8, TNF-α, IL-1β, and IL-6; H-HA of the same concentration does not inhibit L-HA.	TLR2; TLR4	[27]
~200 kDa	10–400 μg/mL	CD44 mediated internalization of HA and covalently linked cargo molecules by tumor lines RT-4 and RT-112/84.	CD44	[28]
234 kDa	50–100 μg/mL	Pharmaceutically pure HA binds to macrophages indirectly by CD44, but does not cause the production of TNF-α, IL-12, CD40, CD86, IL-1β. Binding of HA with macrophages does not lead to the production of TNF-α, IL-12, CD40, CD86, IL-1β. Pharmaceutically pure HA does not cause pneumonia.	CD44	[23]
13 kDa; 97 kDa	10 or 100 μg/mL	HA inhibits the synthesis of NO by LPS-activated macrophages. HA increased the expression of Il-6 mRNA in LPS-activated macrophages and increased the expression of TGF-b1, IL-10, IL-11, Arg1 in untreated macrophages. Low-concentration HA reduced the expression of IL-10 in untreated macrophages.	TLR4	[25]
0.25–0.9 MDa	350 μg/mL	The elevated plasma levels of alanine aminotransferase (ALT) after Con A injection were significantly decreased by pretreatment with high-molecular-weight HAs (780, 900, and 1200 kDa) but not low-molecular-weight HAs (250 and 470 kDa). High-molecular-weight HA (900 kDa) significantly reduced plasma tumor necrosis factor-alpha, interferon gamma, macrophage inflammatory protein 2, and interleukin 4 levels after Con A injection. However, this inhibitory effect on plasma cytokines was not observed with low-molecular-weight HA (250 kDa) pretreatment.	CD44	[29]
0.51 MDa	10 or100 μg/mL	HA inhibits the synthesis of NO by LPS-activated macrophages. HA increased the expression of Il-6 mRNA in LPS-activated macrophages and increased the expression of TGF-b1, IL-10, IL-11, Arg1 in untreated macrophages.	TLR4	[25]
1680 kDa	100 μg/mL	Pharmaceutically pure HA does not bind to CSF-1 BMDM macrophages and has no CD44-mediated interaction. Pharmaceutically pure HA binds to CSF-2 BMDM macrophages indirectly by CD44, but does not cause the production of TNF-α, IL-12, CD40, CD86, IL-1β. Splenic macrophages F4/80+ also bind pharmaceutically pure HA, but splenic DCs do not. Binding of HA with macrophages does not lead to the production of TNF-α, IL-12, CD40, CD86, IL-1β. Pharmaceutically pure HA does not cause pneumonia.	CD44	[23]
1.5–1.8 MDa (<2.5 MDa)	600 μg/mL	H-HA suppressed LPS-induced expression of IL-6, IL-8, TLR4, CXCR1 and increased CD44 expression.	CD44; TLR4	[30]
1.2 MDa	35 μg/mL	H-HA decreased the level of alanine aminotransferase (ALT) in the blood plasma of mice induced by Con A.	CD44	[29]
1.2 MDa	10 and 100 μg/mL	HA has no effect on the NO production of unstimulated RAW 264.7 mouse macrophages. However, HA inhibits the synthesis of NO by LPS-activated macrophages. HA inhibited mRNA expression of TNF-a, Il-6, Il-1b, CCL2 in LPS-activated macrophages (relative to LPS-activated macrophages without HA). HA increased the expression of TGF-b1, IL-10, IL-11, Arg1 in untreated macrophages.	TLR4	[25]
1.54 MDa	150–700 μg/mL	HA+ beta-lapachone suppressed the expression of TNF, PGE2 proteins in LPS-activated macrophages. HA+ beta-lapachone suppressed the PGE2 expression of cartilage explant incubated with LPS.	-	[31]

## 3. Hyaluronic Acid and Oncology

Such optimal properties as low toxicity, high biocompatibility, biodegradation, stability, hydrophilicity, and bioadhesion have allowed the wide use of hyaluronic acid to create delivery systems for antitumor drugs. However, the properties of HA itself in relation to various types of cancer are often not taken into account when using targeted delivery systems. Numerous recent studies have proven the role of hyaluronic acid in the proliferation, invasion, apoptosis, and dormancy of tumor cells [19]. L-HA exhibits pro-inflammatory and pro-angiogenic properties and stimulates tissue damage and cancer metastasis. H-HA is characterized by anti-inflammatory, anti-angiogenic, and antioxidant properties [32,33,34].

Hyaluronic acid is an important component of the extracellular matrix in both normal tissues and tumors. Being the basis of the extracellular matrix, HA can affect various cells of the tumor microenvironment. The role of the tumor microenvironment in carcinogenesis is currently assessed on a par with oncogenes, antioncogenes, and the accumulation of mutations in DNA. There are studies in which a high frequency of driver mutations was detected, but at the same time, the normal structure and functioning of more than a quarter of the cells was preserved [35]. Such studies only confirm the full value of the tumor microenvironment in the origin and development of a tumor. The composition of the tumor microenvironment is represented by fibroblasts, tumor-associated fibroblasts, myofibroblasts, endotheliocytes, pericytes, neutrophilic and eosinophilic leukocytes, etc. [36]. HA fragments obtained by cleavage with hyaluronidase diffuse through tissues and bind to HA receptors on peripheral cells, acting as intercellular signaling molecules. During the progression of malignant neoplasms, the rates of HA biosynthesis and degradation are significantly increased relative to normal tissues. This leads, on the one hand, to increased formation of the extracellular matrix and high-molecular-weight HA, and, on the other hand, to HA fragments. The extracellular matrix, with its high ability to retain moisture, creates optimal conditions for the proliferation and migration of tumor cells [37], and HA fragments stimulate the growth of endothelial cells and angiogenesis [38]. Therefore, HA may act directly on the tumor cell or on a component of the microenvironment.

**Figure 2:**
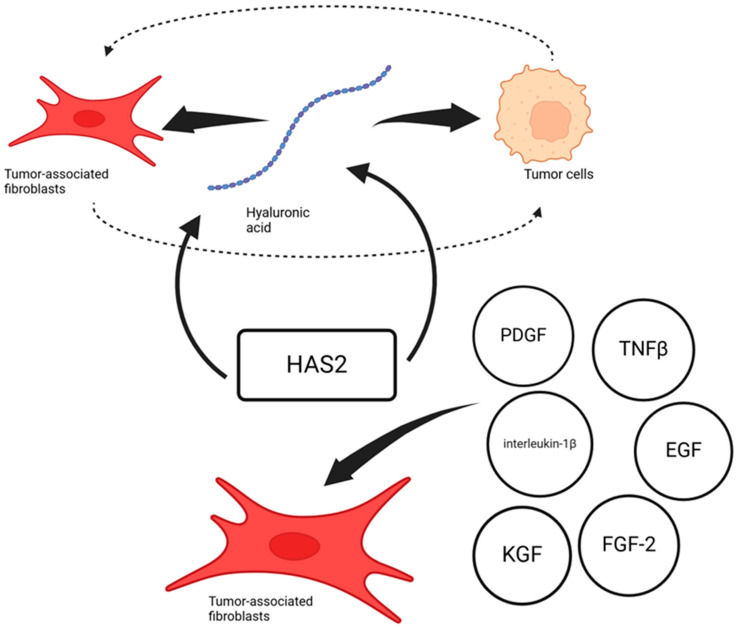
HAS2 expression in tumor tissue and its effects. Created in BioRender. Pashkina, E. (2024) https://BioRender.com/j26x595.

In tumor tissue, an increased level of HA synthesis is noted. When fibroblasts and breast cancer cells were co-cultivated, an increase in the expression of the HAS2 enzyme was noted on fibroblasts, which in turn increased the amount of synthesized HA (Figure 2). In this case, a certain protein synthesized by the cells of the tumor itself, C10 or f118, is indicated as a stimulus for HAS2 hyperexpression [39]. However, this is not the only example of the factors through which tumor cells are able to increase HA synthesis by fibroblasts. HAS2 expression is also regulated by interleukin-1β, FGF-2, PDGF, KGF, EGF, and TGF-β in tumor-associated fibroblasts [40,41].

HA synthesized by tumor-associated fibroblasts themselves has powerful autocrine and paracrine effects in ensuring the migration of both tumor-associated fibroblasts and tumor cells themselves. Hyaluronic acid binds to cell receptors, which additionally promotes the migration and invasion of tumor cells following tumor-associated fibroblasts [42]. The role of HA and its receptors in the motility of tumor cells and their migration has been demonstrated using colorectal cancer and pleural mesothelioma as examples [43,44].

Also, HA in the stroma of solid tumors stimulates the development of PD-L1-expressing macrophages, which reduces the T-cell immune response (via the PD1/PD-L1 pathway) and forms an immunosuppressive microenvironment [45].

## 4. Hyaluronic Acid and CD44

**Figure 3:**
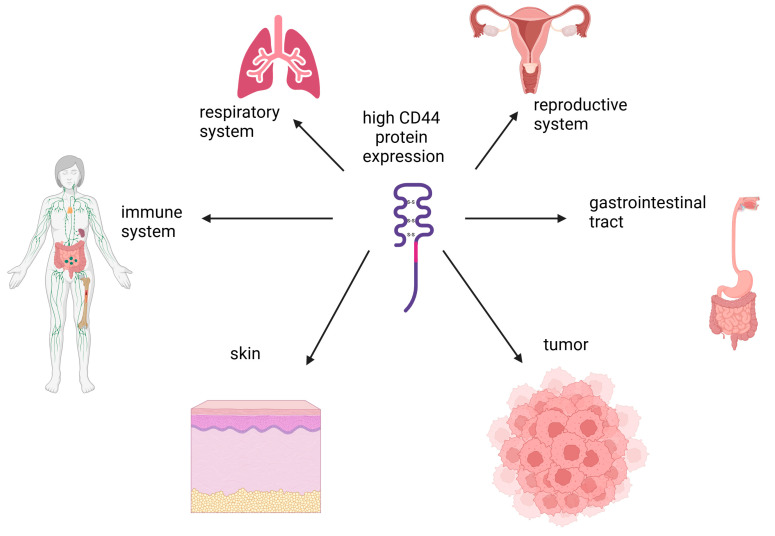
The tissues with high levels of CD44 protein expression. Created in BioRender. Pashkina, E. (2025) https://BioRender.com/z24i114.

CD44 is a type I non-kinase transmembrane protein involved in cell adhesion, migration, and cell–cell interactions [46]. Its role in various biological processes is known, including hematopoiesis, embryogenesis, inflammation, and regeneration. The CD44 gene is located on the short arm of chromosome 11 in humans and on chromosome 2 in mice and has several exons that can undergo alternative splicing, resulting in the formation of variant isoforms of this receptor. Dozens of different CD44 isoforms have been described to date. Among them are the standard isoform (CD44s), the epithelial form (CD44E), and numerous variant isoforms (CD44v) [47,48]. CD44 is expressed ubiquitously in the body, but CD44 expression at the isoform level can vary depending on the tissue type (Figure 3) [49,50,51]. CD44 can bind to various ligands, such as osteopontin, chondroitin, collagen, fibronectin, laminin, and serglycin, but the main ligand for CD44 is HA [48]. The binding activity of CD44 with various ligands, and therefore its functionality, largely depends on the CD44 isoform and its conformational features [47].

To date, a large amount of data has been accumulated indicating the participation of CD44 isoforms in the regulation of signaling pathways (MAPK, Hippo, Hedgehog, PI3K/AKT/mTOR, Twist, HIF, c-Src, Wnt) responsible for the progression of various tumors, the epithelial–mesenchymal transition, invasion and metastasis, as well as resistance to antitumor therapy [52,53,54]. Moreover, many studies demonstrate a correlation between CD44 overexpression and poor prognosis. Thus, the contribution of CD44 to the development of the tumor process, through the above mechanisms, has been described for brain cancer, head and neck cancer, breast cancer, kidney cancer, liver cancer, pancreatic cancer, gallbladder cancer, esophageal cancer, prostate cancer, gastrointestinal cancer, melanoma, squamous cell carcinoma, and sarcoma. A similar role for CD44 has been described for hematological malignancies such as acute lymphoblastic leukemia, acute myeloid leukemia, chronic lymphoblastic leukemia, chronic myeloid leukemia, and multiple myeloma.

The expression of CD44 isoforms and their expression levels often vary depending on the tumor type. Tumor cells can express a different repertoire of isoforms simultaneously, although, as a rule, a predominant isoform stands out among them. For example, in patients with acute myeloid leukemia and glioblastoma, the expression of CD44s prevails, while in patients with colorectal cancer, the expression of CD44v8-v10 prevails in more than 75% of cases [55,56]. Moreover, according to some data, the expression of CD44 isoforms can be plastic, depending on the tumor stage. Thus, some studies demonstrate that, on the one hand, CD44 expression is necessary for tumor initiation and metastatic activity, and on the other hand, suppression of CD44 expression may be beneficial for tumor progression [49]. Such plasticity can be explained by the fact that oncogenic signals are involved in the regulation of variant CD44 exons [57]. As for the specific contribution of different isoforms to tumor development, in most cases, CD44s expression is associated with tumor growth, while CD44v isoforms are more associated with invasiveness and chemoresistance [52].

CD44 actively participates in the process of tumor metastasis. This effect may be due to a parallel with the physiological migration of immature or activated lymphocytes expressing multiple variants of CD44 isoforms [58,59,60,61]. Interestingly, the ability to metastasize can be provided by both CD44s and various variants of CD44v [62,63,64,65,66,67,68]. Moreover, each tumor type apparently has its own pattern of CD44 expression responsible for migration. For example, non-metastatic rat pancreatic cancer cells transfected with CD44v acquired the ability to metastasize. CD44v6 has also been shown to be responsible for lymph node metastasis in intraductal breast carcinoma. In turn, CD44v6 expression by cancer stem cells (CSCs) initiated the metastatic process in colorectal cancer. On the other hand, CD44s has been demonstrated to be responsible for metastatic activity in human lymphoma. However, other studies have demonstrated that CD44 expression is dispensable for tumor metastasis. Deletion of CD44 alleles did not limit in vivo metastatic capacity for MDAY-D2 lymphosarcoma. In contrast, the lack of CD44 in mice impaired osteosarcoma metastasis.

CD44, individually or together with other molecules (CD24, CD133, CD34, and c-Met), can be a key marker for cancer stem cells [59]. Thus, for a number of solid tumors, CD44 binds to the ECM for the maintenance of the stemness of malignant cells [69]. Moreover, intact nuclear CD44/STAT3 signaling has been shown to be critical for reprogramming cancer cells to a CSC phenotype through the transcriptional regulation of c-myc expression and the subsequent self-renewal of CSCs [70]. Interestingly, CSCs may have a unique expression pattern of CD44 isoforms depending on the tumor type. For example, CD44v6 is characteristic of CSCs in colorectal cancer [71], CD44v8-10 in gastric cancer [71], and CD44v3 in head and neck cancer [72]. It is assumed that CSCs are responsible for tumor heterogeneity, which is the main obstacle to the success of antitumor therapy [73]. Taken together, multiple studies in the literature suggest that CD44 is expressed on tumor cells, including cancer stem cells, and is a promising target for targeted therapy in oncology.

Despite the obvious prospects of hyaluronic acid-based drugs as the main ligand for CD44 in antitumor therapy, its effectiveness may depend on which CD44 isoform is expressed in tumor tissue. There is evidence that CD44v isoforms have a significantly reduced ability to bind hyaluronic acid compared to the CD44s isoform. Thus, the binding of CD44v to hyaluronic acid may be limited by steric inconsistency due to the larger size of CD44v, as well as various post-translational modifications of CD44v, such as N-glycosylation and sialylation [74]. It is known that many tumors overexpress the CD44v isoform [75], but there are also tumors with CD44 overexpression [76]. Thus, for tumors overexpressing CD44v, such therapy may be less effective compared to tumors with a predominant expression of CD44s.

## 5. Hyaluronic Acid and Other Receptors of HA

The receptor for hyaluronic acid-mediated motility (RHAMM) is localized inside the cell and is unconventionally exported to the cell surface in response to certain defined stimuli, such as wounding [77]. The mRNA of the RHAMM is present in many tissues and cells, most abundantly in cells of the immune system, gastrointestinal tract, reproductive system, and tumors [50]. There is a direct correlation between the level of RHAMM expression and a worsening prognosis in various types of cancer [78,79,80]. This can be explained by the increased cell migration of tumor cells upon interaction with the RHAMM receptor of low-molecular-weight HA [81,82,83,84]. However, opposite results were also obtained when the genomic reduction of the RHAMM led to the emergence and development of malignant tumors of the peripheral nerve sheaths [85]. Such ambiguous results can be explained by the ability of the RHAMM to bind to numerous proteins and polysaccharides, and its multifunctional nature.

The LYVE1 receptor is located not only on the endothelial cells of the lymphatic vessels, but also on a certain subpopulation of macrophages (LYVE1^+^ macrophages). It is known that LYVE1^+^ macrophages are involved in the internalization and degradation of HA. It was shown that upon depletion of LYVE1^+^ macrophages, there was an excessive accumulation of HA, which had suppressive properties with respect to tumor cells [86]. The possible role of excessive amounts of high-molecular-weight hyaluronic acid in antitumor protection is also discussed using naked mole rats as an example in the work of Xiao Tian et al. [87]. Upon suppression of HAS2 expression or hyperexpression of enzymes that break down HA, it was shown that naked mole rat cells became more vulnerable to malignancy. The authors found that high-molecular-weight hyaluronic acid plays an important role in the activation of the mechanism of early contact inhibition of cells. The activation of early contact inhibition of cells is explained by the inclusion of the HA/CD44/NF2 pathway.

As described above, hyaluronan is able to bind to TLRs, especially TLR4 and TLR2 [24,25,26,27]. HA is considered both as an activating ligand capable of triggering the NF-κB pathway and leading to an increased immune response and the development of inflammation, and as a TLR4 agonist that prevents the receptor from binding to LPS. The question of the role of low-molecular-weight HA as an alarmin remains open at the moment.

Stabilin-2 is the only scavenger receptor capable of recognizing HA [88]. Mice lacking stabilin-2 expression (Stab2KO) have very high HA levels in their circulating plasma, though the mice are physiologically normal in all other aspects [89]. In a study on the role of circulating hyaluronic acid (HA) in a metastatic model, the number of metastatic nodules in the lungs was assessed using Stab2KO mice injected with B16F10 melanoma cells. The results showed that the number of metastatic nodules was lower in Stab2KO mice than in wild-type (WT) mice; significantly fewer cells were observed adhering and interacting in Stab2KO mice compared to WT mice, indicating the importance of circulating HA, or rather the lack thereof, in the process of metastatic cell attachment. Given the potential binding of stabilin-1 to HA-based delivery systems, modification of HA is necessary to prevent accumulation in the liver.

## 6. HA-Based Drug Delivery Systems for Solid Tumors

In the case of HA-based delivery systems, hyaluronic acid, which participates in the construction of the system, can serve as a ligand [90,91,92,93,94]. Hyaluronic acid can bind to various receptors on the cell surface, including CD44, which is expressed at a high level on tumor cells [94]. A conjugate of hyaluronic acid and paclitaxel (Oncofid-P) is currently undergoing clinical trials as a drug for the treatment of bladder carcinoma. In addition, HA can be chemically modified to enable the development of advanced drug delivery systems and biomaterials with enhanced functionalities and targeted applications [95]. Information on clinical trials of HA-based drug delivery systems is presented in Table 2.

Drug delivery systems can enhance drug stability, increase drug solubility, reduce first-pass metabolism, and enable the controlled release of the therapeutic payload [96]. The combination of HA and other molecules included in the drug delivery system not only maintains the ability of hyaluronic acid to target a specific target, but also provides the system with the ability to deliver chemotherapeutic drugs through multiple interactions with tumor microenvironment components of solid tumors. Supramolecular delivery systems based on a hyaluronic acid framework and a macrocycle are one of the promising directions for drug delivery [7,8]. There are various other delivery systems based on hyaluronic acid, including nanoparticles, nanogels, and niosomes [97,98,99,100,101,102]. Additional molecules in the drug delivery system may enhance targeting and/or improve the antitumor properties of the drug. For example, an antitumor effect in vitro and in vivo in a mouse model was obtained using HA nanoparticles with a bilirubin core loaded with doxorubicin. The HA shell allowed the particle to target the CD44-overexpressing cell, and bilirubin enhanced the antitumor effect of the drug and its release in an ROS-containing environment [103].

One of the drawbacks of hyaluronic acid-based drug delivery systems is their poor accumulation in solid tumors due to the superficial penetration depth, low cellular uptake, and non-specific drug release [104]. As described above, hyaluronic acid receptors, including CD44, are widely present in the body and are found not only on tumor cells. Moreover, high expression levels of CD44 on blood cells hinder the transport of the drug delivery system in the bloodstream. On the other hand, the binding of HA to the scavenger receptor may lead to an accumulation of the delivery system not in the tumor, but in healthy tissues. It should also be noted that the dense intercellular matrix in tumor tissue may also hinder drug delivery deep into the tumor. Therefore, various methods are often used to enhance targeted delivery, such as dual targeting, releasing NO for collagen degradation, or HA modification [102,105,106]. Li et al. designed an HA-based drug delivery system that released an immune regulator when located in an immune cell and released doxorubicin in the case of a tumor cell with a lower pH [107]. Zhang et al. [104] prepared an HA nanoparticle conjugated with both NO prodrug (alkynyl-JSK) and doxorubicin prodrug (cis-DOX) for breast cancer therapy. This nanoparticle achieved cascade-enhanced drug delivery efficiency based on a relay strategy of deep tumor penetration, NO uptake by CD44 tumor cells, and HA release of a tumor microenvironment-sensitive drug. A promising direction is the use of nanosized drug delivery systems that are sensitive to the tumor microenvironment and loaded with drugs for tumor immunotherapy [108].

## 7. HA-Based Drug Delivery Systems for Hematological Malignancies

Hematological malignancies are formed from hematopoietic or lymphatic tissues. To maintain therapeutic levels in the bone marrow or lymphatic system, chemotherapeutic drugs require high doses or more frequent administration, which can lead to increased side effects [109,110]. In addition, the bone marrow microenvironment may contain a huge number of tumor stem cells that are resistant to chemotherapy and mediate refractoriness to therapy and disease relapse [111,112].

According to the literature, the expression of CD44, a receptor for hyaluronic acid, has been found in many hematological malignancies [113]. CD44 is involved in the development and progression of hematological malignancies by enhancing apoptotic resistance and invasiveness, as well as regulating bone marrow homing and mobilization of leukemia-initiating cells into the peripheral blood. Also, CD44 is a prognostic marker indicating an increased likelihood of disease relapse [114]. Elevated CD44 expression may serve as a marker of worse prognosis in most hematological malignancies. The expression of CD44 on tumor cells in B-cell acute lymphoblastic leukemia indicates a risk of relapse after the elimination of the primary lesion [115,116]. In acute T-cell lymphoblastic leukemia, high levels of CD44 on cells were noted, and a positive correlation was observed with the development of infiltration of the internal organs of patients [117]. The role of the NOTCH1-MYC-CD44 axis in the risk of relapse in T-cell acute lymphoblastic leukemia associated with the persistence of leukemia-initiating cells was also noted [118].

Despite numerous data on the role of CD44 in tumors of hematopoietic and lymphoid tissues, CD44 has been rarely considered as a target for targeted therapy for this type of pathology [118]. Thus, nanoparticles were created for the treatment of non-Hodgkin’s lymphomas; the outermost layer of the particle consisted of hyaluronic acid, covalently conjugated with antibodies to CD20, which provided dual targeting to tumor cells [119]. To provide antitumor action, the nanoparticles were loaded with siRNA. It was found that the introduction of the HA-based drug delivery system induced apoptosis of target cells and prevented the proliferation of blood cancer cells both in cell culture and in animal models of non-Hodgkin’s lymphoma. A similar approach was used in a similar system, only the authors performed triple targeting (hyaluronic acid and two antibodies to surface markers) [120]. A different approach was used by another group of authors, who took doxorubicin-loaded nanoparticles as a basis [121]. The particle surface was modified with hyaluronic acid for targeted delivery. It was shown that doxorubicin encapsulated in hyaluronic acid nanoparticles cross-linked with lipoic acid (LACHA-DOX) mediated the highly effective and targeted inhibition of human tumor cells, including human multiple myeloma LP-1 and human acute myeloid leukemia AML-2, during xenotransplantation of cells into nude mice. Duvelisib-loaded nanoparticles coated with hyaluronic acid have demonstrated their safety and efficacy in models based on CD44-positive cell lines of hematological malignancies [122]. Silver-based nanoparticles coated with hyaluronic acid enhanced ROS production in leukemia cells and induced tumor cell apoptosis [123]. Therefore, the results indicate the effectiveness of targeting HA-based delivery systems for the treatment of blood cancers.

## 8. Conclusions

HA has optimal properties such as low toxicity, high biocompatibility, biodegradability, and stability, which allows it to be widely used in the creation of drug delivery systems. Many tumors, including various solid and blood cancers, highly express receptors for hyaluronic acid. As the present studies indicated, HA-based drug delivery systems have shown their effectiveness and safety. However, the use of targeted therapy on HA-based delivery systems does not always allow for achieving the desired therapeutic effect due to a low accumulation in the tumor. Moreover, in most cases, the development of new HA-based delivery systems is directed towards the treatment of solid tumors, while hematological malignancies often remain without due attention, despite the high expression of CD44 on blood cancer cells. Therefore, more advanced and multi-component drug delivery systems are needed to eradicate various types of cancer with greater efficacy and selectivity and less toxicity, and their efficacy and safety should be confirmed by clinical trials.

## Figures and Tables

**Table 2:** Clinical trials of HA-based drug delivery systems.

Drug Delivery System	Tumor	ID Number
Oncofid-P (paclitaxel–hyaluronic acid conjugate)	Bladder Carcinoma in Situ	NCT04798703 NCT05024773
Oncofid-P (paclitaxel–hyaluronic acid conjugate)	Non-Invasive Papillary Carcinoma of Bladder	NCT04661826
FOLFIRI versus FOLF(HA)iri (the FOLFIRI regimen with the “HA-Irinotecan”) regimen	Metastatic Colorectal Cancer	NCT01290783
FOLF(HA)iri with cetuximab	Metastatic Colorectal Cancer	NCT02216487

## Data Availability

No new data were created or analyzed in this study.
